# Radiation-associated angiosarcoma of the breast: radical resection technique of the entire radiation field

**DOI:** 10.1177/17588359251317842

**Published:** 2025-02-18

**Authors:** Johannes Tobias Thiel, Michael Bauer, Adrien Daigeler, Vladyslav Kavaka, Jonas Kolbenschlag, Dominik Steiner, Anna Storz, Sebastian Hoffmann

**Affiliations:** Department of Hand, Plastic, Reconstructive and Burn Surgery, BG Trauma Center Tübingen, University of Tübingen, Schnarrenbergstrasse 95, Tübingen 72076, Germany; Department of Hand, Plastic, Reconstructive and Burn Surgery, BG Trauma Center Tübingen, University of Tübingen, Tübingen, Germany; Department of Hand, Plastic, Reconstructive and Burn Surgery, BG Trauma Center Tübingen, University of Tübingen, Tübingen, Germany; Department of Hand, Plastic, Reconstructive and Burn Surgery, BG Trauma Center Tübingen, University of Tübingen, Tübingen, Germany; Department of Hand, Plastic, Reconstructive and Burn Surgery, BG Trauma Center Tübingen, University of Tübingen, Tübingen, Germany; Department of Hand, Plastic, Reconstructive and Burn Surgery, BG Trauma Center Tübingen, University of Tübingen, Tübingen, Germany; Department of Hand, Plastic, Reconstructive and Burn Surgery, BG Trauma Center Tübingen, University of Tübingen, Tübingen, Germany; Department of Hand, Plastic, Reconstructive and Burn Surgery, BG Trauma Center Tübingen, University of Tübingen, Tübingen, Germany

**Keywords:** breast, breast-conserving therapy, chemotherapy, radiation-associated angiosarcoma, surgical technique

## Abstract

**Background:**

Radiation-associated angiosarcoma of the breast (RAASB) is a rare secondary angiosarcoma that typically develops subsequent to breast-conserving therapy for breast cancer. The parameters of the resection width and depth remain the subject of considerable controversy. More recent data indicate that radical resection of the complete radiation field at the thorax is associated with improved local control and survival.

**Objectives:**

The present study investigates the radical resection technique of the entire radiation field and subsequent defect coverage in RAASB, as well as the medium-term follow-up.

**Design:**

Monocentric, retrospective, and non-comparative study.

**Methods:**

From January 2017 to January 2024 a total of 10 patients with RAASB were treated at our hospital. The radical resection technique was employed in the treatment of all patients, encompassing the entire radiation field. Three patients received local flaps (two of whom received vertical and transversal rectus abdominis muscle flaps and one received a local random pattern flap), while the remaining seven were treated with split-thickness skin grafts for defect coverage.

**Results:**

The median age at initial diagnosis of breast cancer was 59.3 ± 9.41 years, while that of RAASB was 66.2 ± 8.32 years. The median latency period between the start of irradiation of the chest wall and the initial presentation of RAASB was 6.5 ± 3.08 years. The cumulative median total radiation dose was 57.23 ± 8.34 Gray (cumulative Gray) in 9 of the 10 patients. The overall survival (OS) was 80% in the cohort, with a median follow-up period of 40.0 ± 27.96 months. Three patients exhibited local relapses following radical resection, with two of these patients ultimately succumbing to their condition.

**Conclusion:**

Patients with RAASB may benefit from a radical resection of the entire radiation field. Despite the relatively mutilating nature of the procedure, the radical resection technique may have the potential to reduce the rate of local recurrence and prolong OS.

## Introduction

Surgical resection with negative margins represents the standard of care for a rare radiation-associated angiosarcoma of the breast (RAASB), and it is the preferred treatment option for all resectable sarcomas.^1–6^ The occurrence of RAASB is uncommon, defined as the development of a sarcoma in a previously treated field of radiotherapy with a minimum latency period of 3 years.^7^ Consequently, it can be regarded as a secondary form of angiosarcoma (AS). The median time to the radiological appearance of RAASBs is 6–8 years after radiation of the breast as a part of a multimodal therapy regimen for breast cancer.^1,7,8^ In recent years, there has been a marked increase in the absolute number of diagnosed RAASBs in concordance with the number of patients undergoing breast-conserving surgery (BCT) and adjuvant radiotherapy (RT).^3,9–14^ In the United States, the estimated absolute risk of radiation-associated RAASBs is 7–10 per 100,000 person-years post-BCT.^9,14^ The most recent data indicate a notable rise in the incidence of the condition over the last 30 years, with an estimated 30 cases of RAASB per 100,000 person-years after BCT.^14^ A significant challenge for the surgeon performing the initial treatment is the multifocal, typically cutaneous growth of radiation-induced ASs.^2,3,10^ This is further complicated by the inability of imaging techniques such as CT or contrast-enhanced MRI to fully visualize the tumor margins in cutaneous lesions prior to surgery.^15^ Consequently, the treating surgeon must rely on clinical estimation to determine the extent of resection prior to the procedure.^10^ Earlier attempts like preoperative tumor mapping using circular trial incisions demonstrated no advantage and have since been abandoned by the majority of centers, as has been the case with ours. Furthermore, the depth of resection is also a topic of debate.^16^ As is the case with all rare diseases, the primary issue is the lack of studies and evidence. To date, there are no prospective randomized studies regarding RAASB. Most of the evidence is based on case reports and case series. In this study, we present our retrospective monocentric findings for the radical surgical approach as recommended by Li et al.,^2^ along with our resection technique and plastic reconstruction concepts that we established for RAASBs at our sarcoma center.^2^

## Methods

### Patient and study design

Following approval from the institutional ethics committee (440/2024BO2; July 17, 2024), data for all patients with RAASB who underwent a radical resection of the entire radiation field at our university hospital between January 2017 and January 2024 were retrospectively reviewed. A written declaration of consent was obtained from all subjects participating in the study and the published patient images. Patients with primary breast AS or radiation-associated sarcomas other than AS were excluded from the study. The commencement date of the patient’s radiotherapy treatment for primary breast cancer was utilized to calculate the time interval between the conclusion of radiotherapy and the diagnosis of RAASB. This approach was adopted due to the unavailability of the end dates for the radiotherapy treatment. The data of one patient (Patient 3), who received an RT at an outside institution, was not available in our records.

The primary outcome variables were defined as follows: Cumulative incidence (CI) of local recurrence, CI of distant metastases, overall survival (OS), and postoperative complications after radical resection therapy of RAASB. Postoperative complications were classified according to the Clavien-Dindo system. Local recurrence was defined as the reappearance of the tumor in the ipsilateral breast. Distant metastasis was defined as the reappearance of the tumor in any other location, including the contralateral breast, chest wall, and adjacent abdominal wall. OS was defined as the time from the date of initial RAASB surgery to the date of death.

### Surgical technique(s)

#### Soft tissue resection and split-thickness skin graft

**Figure 1.**
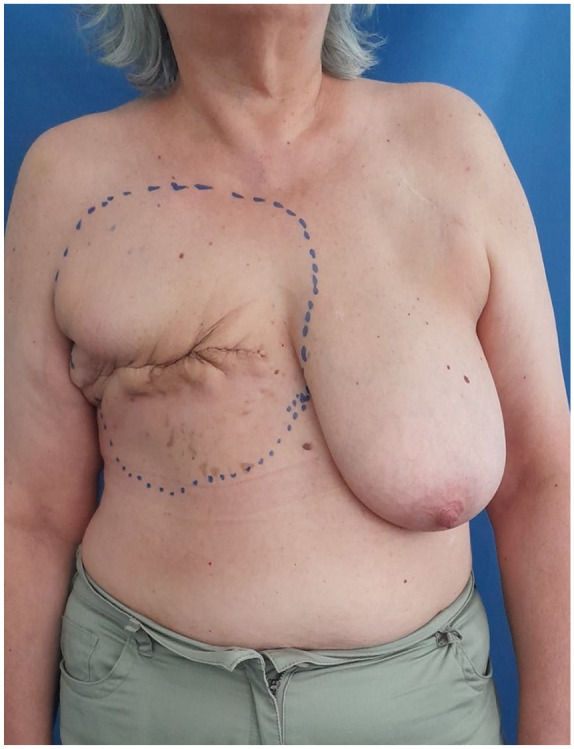
Pre-operation markings of the radiation field. In this instance, the mastectomy with the RAASB tumor mass was performed outside our hospital. The initial resection concept was extended from the more radical, previously described variant with the objective of improving local control and disease-specific survival. RAASB, radiation-associated angiosarcoma of the breast.

**Figure 2.**
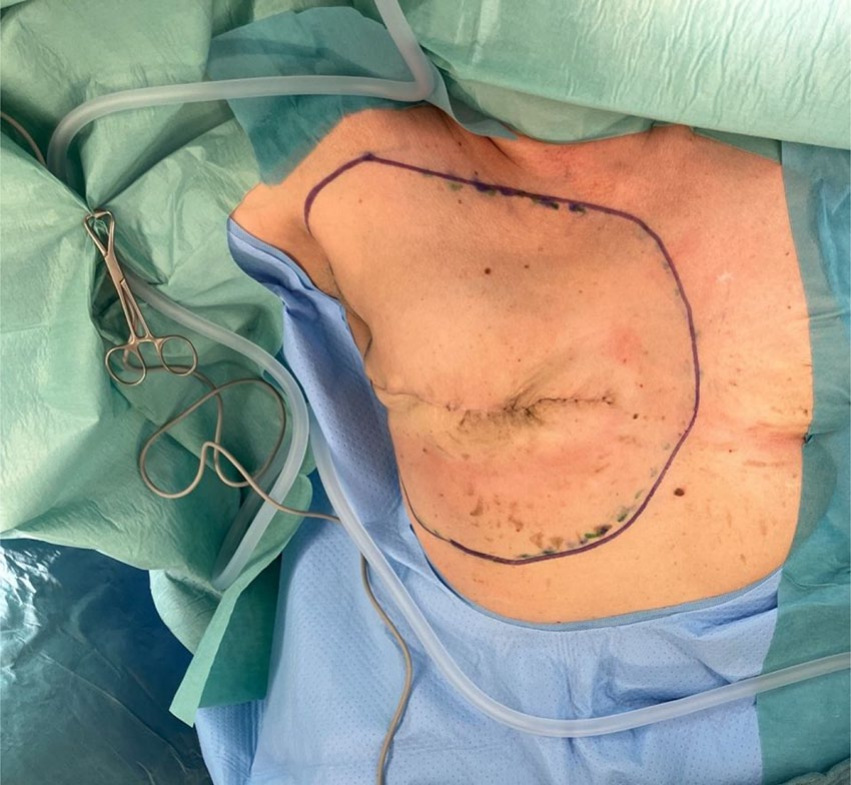
The final resection is conducted on the patient in a supine position with the affected extremity positioned externally.

**Figure 3.**
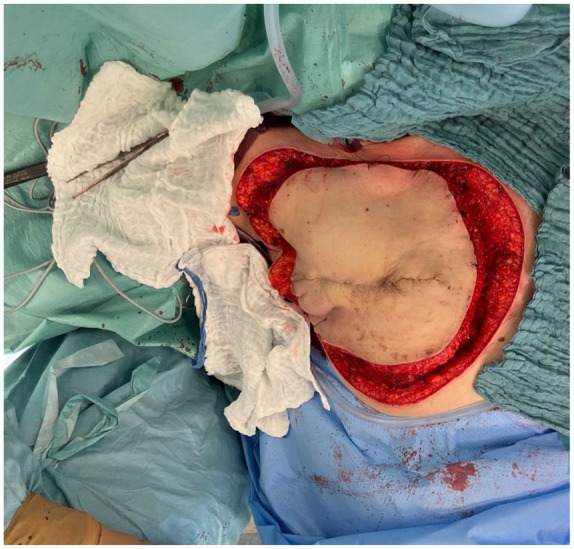
A completely dissected specimen is presented herewith. It should be noted that the dissection is strictly subfascial in the case of the purely cutaneous RAASB. RAASB, radiation-associated angiosarcoma of the breast.

**Figure 4.**
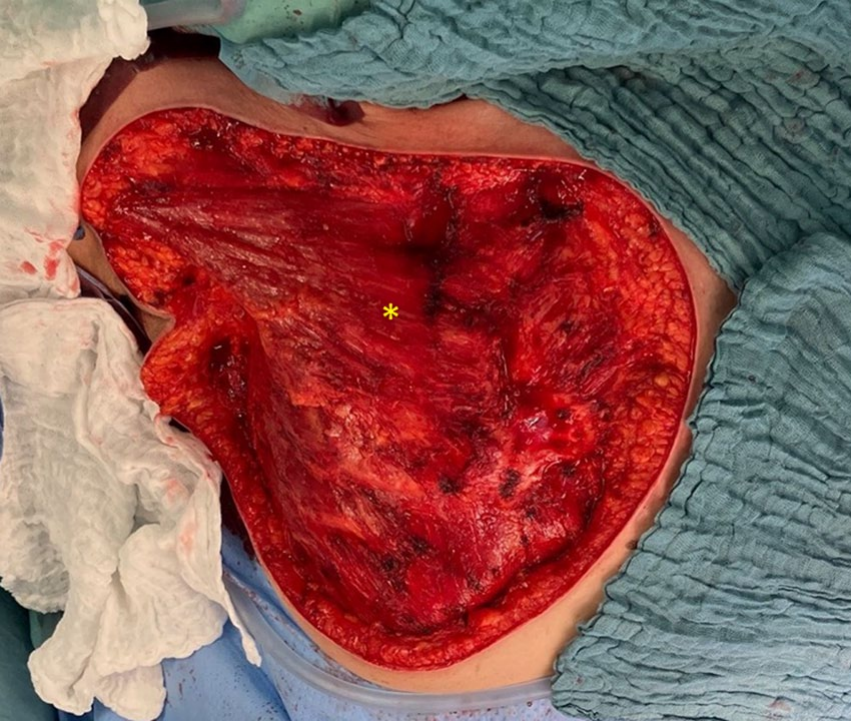
The image depicts the site following resection. The remaining pectoral muscles, indicated by the asterisk (*), are clearly visible.

**Figure 5.**
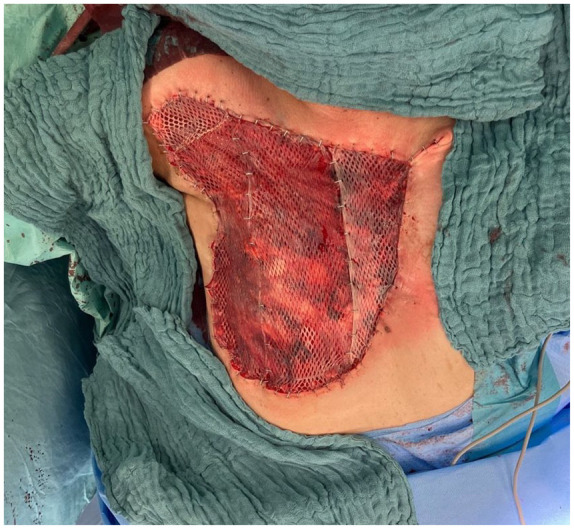
Following the implementation of wound reduction and split-thickness skin grafting procedures, a vacuum dressing with 80 mmHg suction is then applied.

All patients were presented for review by the interdisciplinary sarcoma board of the University of Tuebingen prior to undergoing surgery. The preoperative diagnostics were completed by staging examination. Prior to the surgical procedure, the standard radiation field was delineated, and the boundaries of the planned resection were definitively established. The cranial border was delineated by the clavicle, lateral border by medial axillary line, the caudal border was marked by the xiphoid process and the fifth or sixth rib, and the medial border by the lateral edge of the contralateral sternum (Figure 1). In the case of a purely cutaneous AS (the majority of the cohort in the literature and of ours), complete resection of the skin, subcutaneous fatty tissue, and, if necessary, the remaining mammary gland tissue is performed using monopolar electrocautery, taking along all muscle fascia (subfascially) in the radiation field in the area of the anterior thoracic wall (Figure 2). Subsequently, the wound edges are retracted, and the wound size is reduced using 3-0 polydioxanone running intradermal sutures to fixate the skin edges to the chest wall (Figures 3 and 4). Ultimately, the soft tissue defect of the hemithorax is addressed by utilizing split-thickness skin graft (STSG) from the thigh with a dermatome in 0.2 mm thickness, employing the mesh technique in a size ratio of 1:1.5 (Figure 4). Subsequently, the STSG is stapled, and a vacuum dressing is applied, with suction maintained at 80 mmHg continuously. The vacuum dressing is then left in place for 5 days, the metal staples are removed after another 2 days (Figure 5).

**Figure 6.**
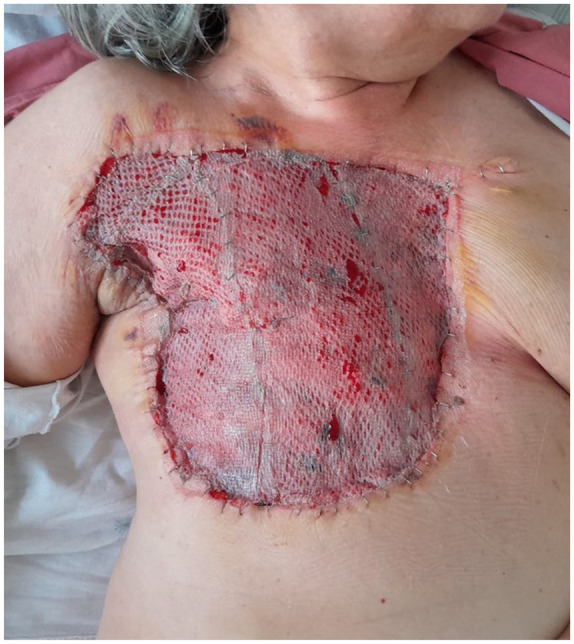
The split-thickness skin graft is in the process of healing on the fifth postoperative day, following the removal of the vacuum dressing.

#### Deep resection and defect coverage with pedicled combined vertical and transversal rectus abdominis muscle flap

In the event of additional involvement of the musculature of the anterior thoracic wall, a deep resection is performed, taking along the affected musculature and, if needed, the rib periosteum. The resulting defect is then covered using a pedicled combined vertical and transverse rectus abdominis muscle flap (VRAM/TRAM flap or anchor flap). In the case of bony infiltration of the thoracic wall, the thoracic wall is also resected, and the resulting bony defect is reconstructed using a large pore polypropylene mesh. Subsequently, coverage is achieved with a pedicled anchor flap from the abdomen with VRAM/TRAM flaps. At our clinic, we prefer local pedicled flaps for initial defect coverage for deep defects, as this allows us to utilize free flaps in the event of local tumor recurrences.

### Statistical analysis

A descriptive statistic of the presented data was performed using GCM Medico (CompuGroup Medical Deutschland AG, 56070 Koblenz, Germany) and JASP version 0.19.1 (Eric-Jan Wagenmakers, Department of Psychological Methods, University of Amsterdam, Nieuwe Achtergracht 129B, Amsterdam, The Netherlands). Descriptive statistics were expressed as the mean and standard deviation.

### Study reporting

The reporting of this study conforms to guidelines for surgery techniques from the SUPER 2023 checklist.^17^

## Results

A total of 10 patients with RAASB were identified within the specified time frame between January 2017 and January 2024. The median age at initial diagnosis of breast cancer was 59.3 ± 9.41 years and of RAASB 66.2 ± 8.32 years. The median latency period between the start of irradiation of the chest wall and the initial presentation of RAASB was 6.5 ± 3.08 years. In 9 of 10 patients, the cumulative median total radiation dose was 57.23 ± 8.34 cGy. Basic characteristics of each patient can be found in Table 1. Breast cancer and RAASB-related characteristics and postoperative outcomes are presented in Tables 2 and 3, respectively. Two patients received VRAM/TRAM flaps while one patient received a local random pattern flap from the back. The remaining seven patients were treated with STSG. The overall survival (OS) and disease-specific survival (DSS) is 80% in our cohort in a follow-up period of 40 ± 27.96 months. One patient had a recurrent tumor 9 months after radical resection of RAASB (this was her combined sixth recurrence) and lung metastasis and died finally 11 months after radical resection and STSG. The other patient died after a total of four local recurrences (three local relapses occured after radical resection) and 20 months after radical surgery. A third patient exhibited a recurrence at the former cranial resection margin in November 2024, 4 years after radical resection. The recurrence was resected with wide negative margins, and STSG was used to cover the defect.

**Table 1.** Basic characteristics of RAASB patients who received radical resection of the entire radiation field.

Patient	Age at diagnosis of RAASB (in years)	Sex	Localization	Comorbidities	Smoking status	Second malignancy
1	63	Female	Right breast	Asthma	Yes	No
2	61	Female	Right breast	Arterial hypertension, glaucoma bilaterally, GERD	No	No
3	73	Female	Right breast	Arterial hypertension, bronchial asthma, Lumbar herniated disk, Hypercholesterolemia	Yes	Carcinoma of visceral vessels
4	69	Female	Right breast	Hypothyroidism, arterial hypertension, history of pulmonary embolism, and DVT	N/A	No
5	60	Female	Left breast	None	No	No
6	56	Female	Right breast	MGUS, Glaucoma	No	No
7	85	Female	Right breast	Bronchial asthma, arterial hypertension, hyperthyroidism, Type 2 diabetes mellitus	N/A	No
8	61	Female	Right breast	Hypothyroidism, type 1 diabetes mellitus	Yes	No
9	65	Female	Left breast	Arterial hypertension, varicose veins, COPD, sleep apnea, depression, type 2 diabetes mellitus	Yes	N/A
10	69	Female	Left breast	Liver hemangioma, pseudomembranous colitis, arterial hypertension, hypothyroidism	No	No

COPD, chronic obstructive pulmonary disease; DVT, deep vein thrombosis; GERD, gastroesophageal reflux disease; MGUS, monoclonal gammopathy of undetermined significance; N/A, not applicable; RAASB, radiation-associated angiosarcoma of the breast.

**Table 2.** Breast cancer and RAASB-related characteristics.

Patient	Age at breast cancer diagnosis (in years)	Year of breast cancer diagnosis	Interval from BCT to RAASB diagnosis (in years)	Start of radiotherapy (year)	Interval from radiotherapy to RAASB diagnosis (in years)	Year of RAASB diagnosis	Type of breast cancer surgery	Cumulative radiation dose (cGy)	Interval from RAASB diagnosis to first surgery in Domo (in month)	Size of the RAASB during resection of the entire radiation field (in cm)
1	63	2020	3	2021	2	2023	BCT	50.05	4	Prophylactic resection, no tumor
2	61	2011	10	2011	10	2021	BCT	70	5	2.4 × 2.1 × 0.9
3	73	2011	7	2011	7	2018	BCT	N/A	5	Multiple lesions
4	69	2013	6	2013	6	2019	BCT	59	20	8
5	60	2007	8	2008	7	2015	BCT	64	1	Multiple lesions
6	56	1999	8	2000	8	2008	BCT	60	99	No tumor
7	85	2013	5	2013	5	2018	BCT	50.4	1	14 × 7 × 4
8	61	2005	13	2005	13	2018	BCT	59.4	9	Multiple lesions
9	65	2018	5	2019	4	2023	BCT	42.2	2	12
10	69	2008	11	2013	6	2019	BCT	60	3	Multiple lesions

BCT, breast-conserving therapy; cGy, cumulative Gray; RAASB, radiation-associated angiosarcoma of the breast.

**Table 3.** Surgical characteristics and postoperative outcome.

Patient	Year of radical resection	Resectate size of irradiation field (in cm^2^)	Negative margins	(Neo-) Adjuvancy	Defect coverage	Postoperative complications (Clavien-Dindo Score)	Type of postoperative complications	Recurrent RAASB at time of presentation in our hospital	Number of relapses *before* radical surgery	Number of relapses *after* radical surgery	Follow-up (in month)	Survival
1	2023	408,3	Yes	No	STSG	No	N/A	No	0	0	11	Yes
2	2021	578,85	Yes	Paclitaxel (adjuvant)	STSG	No	N/A	Yes	1	0	34	Yes
3	2020	500	Yes	No	STSG	II	Wound healing disorder STSG	Yes	4	0	49	Yes
4	2020	322,5	Yes	No	STSG	No	N/A	Yes	2	1	61	Yes
5	2017	461,25	Yes	No	STSG	No	N/A	Yes	5	1^a^	11	No
6	2017	1000	Yes	No	VRAM/TRAM	No	N/A	Yes	4	0	86	Yes
7	2018	N/A	Yes	No	Random pattern local flap	IIIb	Wound dehiscence	No	0	0	78	Yes
8	2018	480	Yes	Radiation 65 Gy (adjuvant)	VRAM/TRAM	IIIb	Partial flap-necrosis	Yes	1	3	20	No
9	2023	626,04	Yes	No	STSG	No	N/A	No	0	0	14	Yes
10	2020	N/A	Yes	No	STSG	II	Wound healing disorder STSG	No	0	0	47	Yes

a Died because of recurrent disease of lung metastasis and local recurrence.

STSG, split-thickness skin graft.

**Figure 7.**
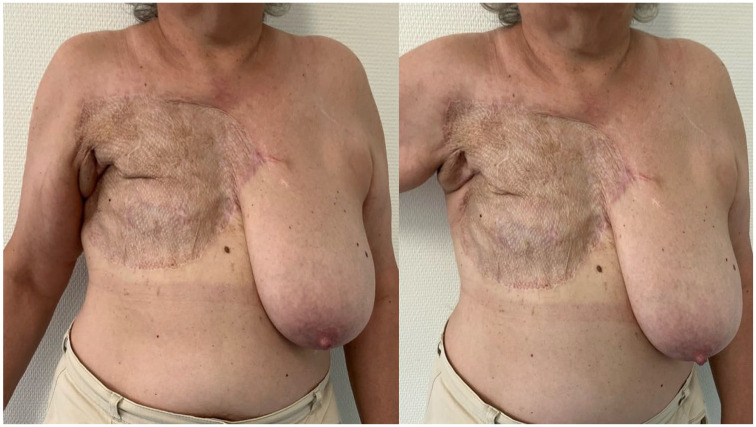
The postoperative result was evaluated 3 months following the initial procedure.

**Figure 8.**
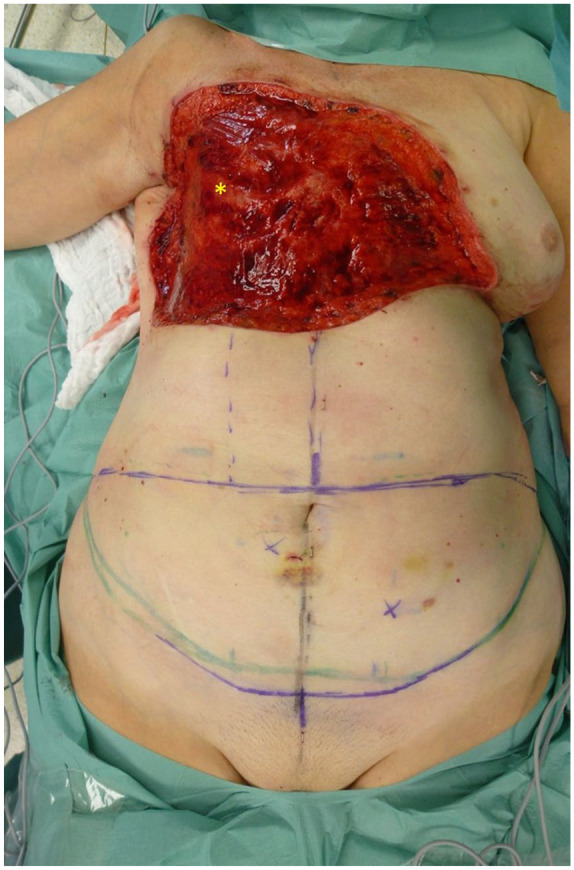
Defect of the right thoracic wall after resection of RAASB with infiltrated *pectoralis* major muscle. It should be noted that some of the ribs are exposed (asterisk). RAASB, radiation-associated angiosarcoma of the breast.

**Figure 9.**
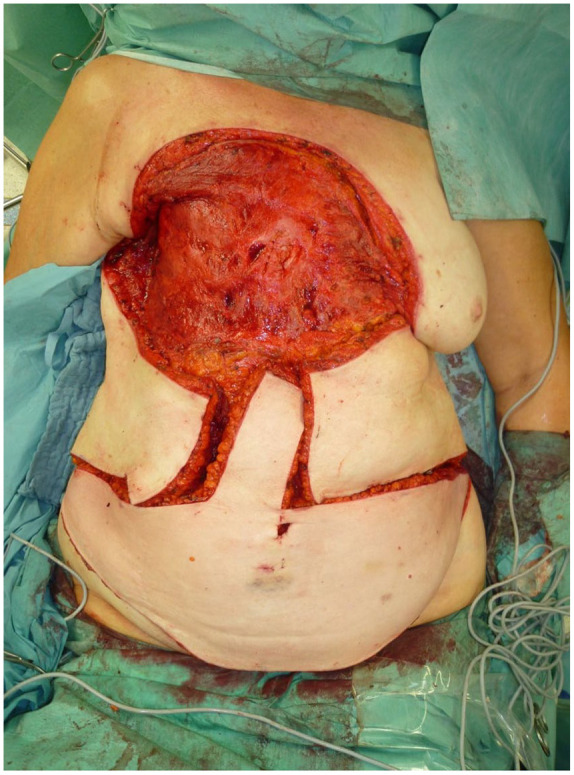
Raised pedicled combined VRAM/TRAM-flap. VRAM/TRAM, vertical and transverse rectus abdominis muscle flap.

**Figure 10.**
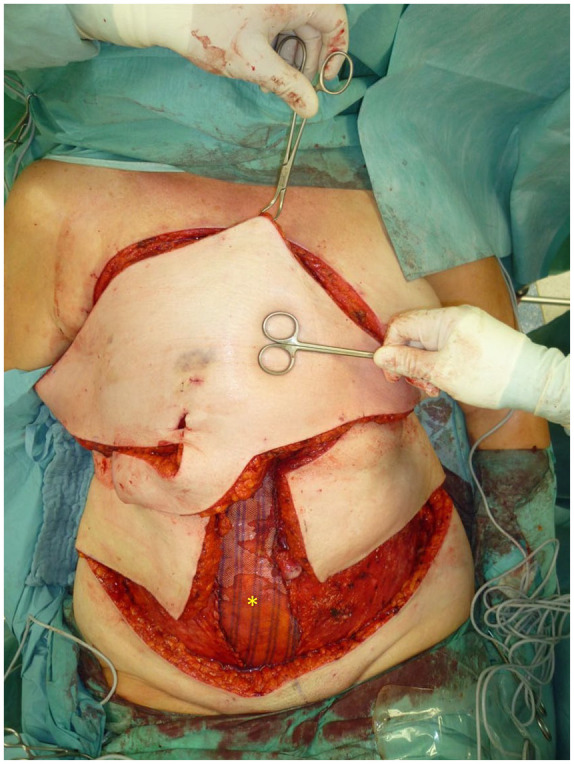
Flap rotated counterclockwise into the defect. The donor defect on the right abdomen was reconstructed with a polypropylene mesh (asterisk (*)).

**Figure 11.**
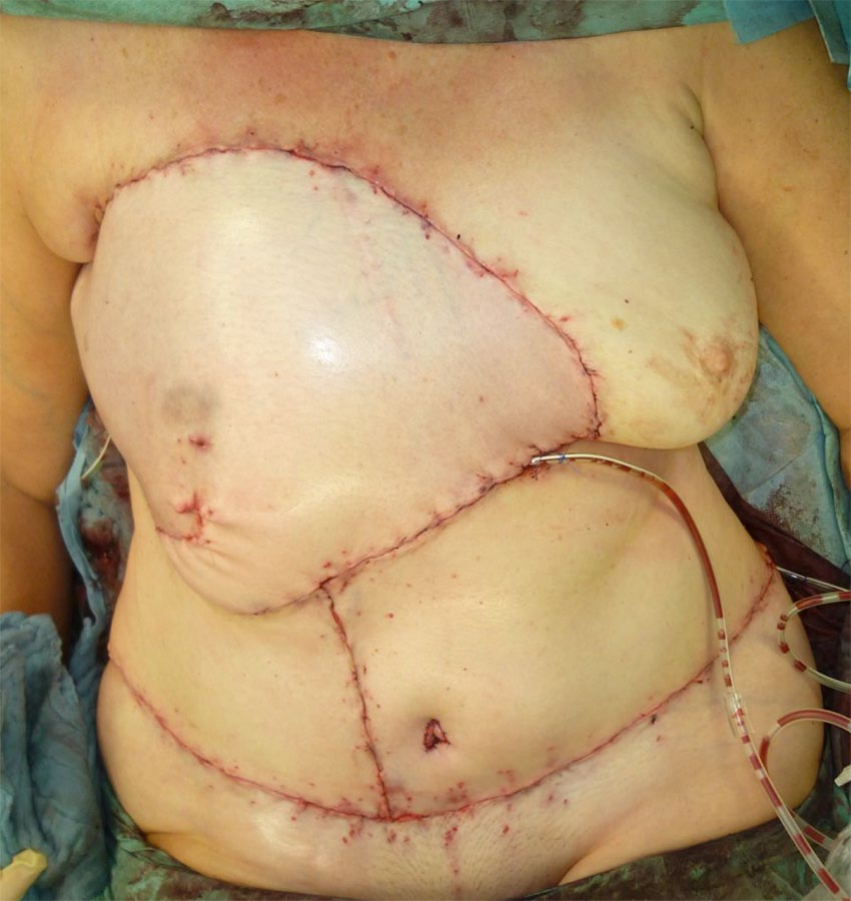
Complete sewn in and well vascularized combined VRAM/TRAM-flap. VRAM/TRAM, vertical and transverse rectus abdominis muscle flap.

**Figure 12.**
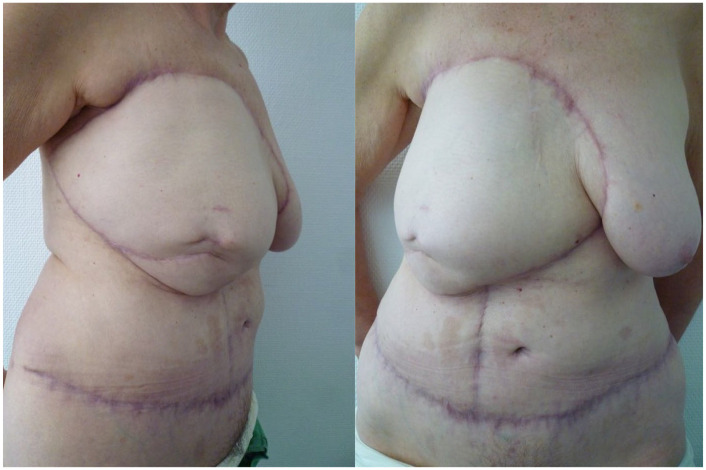
Postoperative result after 3 months.

## Discussion

RAASBs are predominantly cutaneous and locally aggressive sarcomas, exhibiting historically elevated local recurrence rates and poor long-term survival rates.^2,14,18–23^ In this retrospective study, we present our experience with the radical resection technique that has been employed at our sarcoma center since 2017. Due to the new start initiated by the acting clinic director in 2017, our department became part of the Sarcoma Center at the University of Tübingen. Since then, the relevant expertise has been applied clinically, and experience gained. The preliminary results, particularly with regard to local recurrence, and overall and disease-specific survival, are encouraging, although the follow-up period remains relatively brief. Despite the encouraging indications of improved OS and lower local recurrence rates in the literature, communicating the necessity of such a large and, in comparison to a simple mastectomy, mutilating procedure to both the local sarcoma board and patients remains a significant challenge.^2,3^

As demonstrated, either STSG or (myo-)cutaneous flaps are required for defects measuring approximately 30 × 30 cm on the thoracic wall. The corresponding techniques have been described in detail. However, 40% of the cohort developed postoperative wound healing disorders, necessitating reoperation in 2 out of 10 patients.

Some studies have demonstrated enhanced local control and improved OS with radiation (neoadjuvant, adjuvant, or as a sole therapy without surgical resection) in RAASB.^3,24,25^ This stands in contrast to the prevailing wisdom regarding the re-irradiation of tumors that have been caused by radiation. However, these groups have reported favorable outcomes. In a retrospective study by Donovan et al., nine patients with RAASB underwent radical surgery and immediate hyperfractionated accelerated radiotherapy (HART).^3^ Radical resection of the chest wall and en bloc mastectomy were performed on the patients. Following this, radiotherapy was administered with 4500 cGy in 45 fractions. In the course of the treatment, one of the nine patients developed local recurrence and metastasis, while one patient died from other causes. The remaining seven patients were alive with no evidence of recurrent disease. Seven cases of mild skin toxicity were reported during treatment. One patient developed chronic wound healing complications, which eventually resolved, and one patient developed asymptomatic radiation osteitis of a rib. Nevertheless, the median follow-up period was found to be shorter at 19 months (range 3–41 months) than in the present study (40 months) or the subsequent one (60 months). In a retrospective study by Palta et al., 14 patients with RAASB were observed with regard to HART in combination with and without surgery. Progression-free survival rates for the 14 patients at 2 and 5 years were 71% and 64%, respectively. The overall and cause-specific survival rates (DSS) were both 86% at 2 and 5 years.^24^ The OS and DSS appear to be analogous to our own in both studies, although the resection technique is not precisely delineated in the study of Palta et al. and the follow-up periods differ. Local control was highest in the study by Donovan et al. (one of nine had local recurrence), followed by our study (3 of 10 patients had local recurrence). The study by Palta et al. came in last, with a total of 5 recurrences (of 14 patients) after HART (median 1 month). However, the use of re-irradiation without a well-defined complication profile remains a topic of debate. Given the favorable outcomes observed with a radical R0 resection of the entire radiation field, adjuvant radiotherapy is not a standard recommendation in our practice. Notwithstanding, one female subject underwent an adjuvant re-irradiation procedure in the course of our study. As previously indicated, two female subjects exhibited a local recurrence relatively soon after undergoing radical resection. At least one of these subjects also developed lung metastasis, which resulted in death after 11 and 20 months of radical resection, respectively. As demonstrated in Tables 1 to 3, both patients had already experienced at least one recurrence of RAASB prior to undergoing treatment at our institute (see Patients 5 and 8). We hypothesize that radical resection at the initial manifestation of RAASB results in a higher OS and lower local recurrence rates, but we cannot prove this based on our data. In the study by Li et al. from 2017, disease-specific survival was not prolonged by a radical resection of recurrences.^2^ Their data supported radical resection only for initial tumors. Their largest retrospective comparative study to date showed that 5-year survival in the radical resection group (*n* = 38; complete resection of the entire irradiated field) was 86% compared to 46% in the non-radical group (*n* = 38; simple mastectomy or wide excision).^2^ However, a significantly higher proportion of patients in the “radical group” underwent additional chemotherapy (58% vs 22%, *p* < 0.01). It can be argued, therefore, that radical and mutilating surgery should only be considered as a last resort for treating relapses. However, as already mentioned, the level of evidence supporting all existing studies is relatively low. Consequently, our patients are informed accordingly and advised that radical resection is the best option for achieving medium- to long-term results despite its limitations in recurrent cases.

A systematic review from 2014 looked at 74 articles with data from 222 patients. In these patients, the 5-year OS was 43% and the 5-year local recurrence-free interval (LRFI) was 32%.^11^ Of all patients, 68% received conservative surgery alone, 17% conservative surgery and radiotherapy, and 6% conservative surgery and chemotherapy. The remaining 9% received primary treatment without surgery. Conservative surgery with radiotherapy resulted in a better 5-year LRFI of 57% compared to 34% with surgery alone (*p* = 0.008). It was not possible to analyze the other treatment groups.

In a recent retrospective study from 2024, the results of surgery plus neoadjuvant chemotherapy (NACT; *n* = 13, predominantly treated with paclitaxel) were compared with the results of surgery without chemotherapy (*n* = 22).^26^ In the group treated with NACT, no metastases or deaths were observed at a median follow-up of 41 months, and only one recurrence occurred after 6.5 years. OS was significantly increased in the NACT group (100% vs 56.1%, *p* = 0.016).

There are several limitations to this study. It is important to note that this is a retrospective, descriptive study. In addition, the sample size is relatively limited, which reduces the study’s statistical power. Furthermore, the follow-up period is relatively short, which increases the likelihood of random errors.

Notwithstanding these constraints, our data, along with those from other research centers, indicate that radical resection of the entire radiation field may potentially lead to superior local control and OS in RAASB. Consequently, upon confirmation of the diagnosis of RAASB, radical subfascial resection of the skin and soft tissue of the entire irradiation field is performed at our hospital, typically followed by defect coverage with STSG. In cases involving muscle infiltration and/or bony thoracic wall involvement, a cross-compartment resection of the entire radiation field is performed at our institution, along with a reconstruction utilizing VRAM/TRAM flaps. In the curative approach, (neo-)adjuvant radio-/chemotherapy plays a subordinate role at our institution. Finally, chemotherapy and/or radiotherapy are most likely to be considered by us in the palliative setting.

## Conclusion

A comparison of older data with more recent and our data indicates that radical resection of the entire radiation field in RAASB patients appears to improve OS when compared with data from less radical surgery. However, recent data suggest that NACT in combination with surgical resection may offer a distinct advantage in RAASB patients, despite the absence of reports on radical resection of the entire radiotherapy field. Further studies are required to ascertain whether radical resection of the entire radiation field in combination with NACT with paclitaxel could be a decisive factor in terms of OS and local control in a multimodal setting.

## Supplemental Material

sj-docx-1-tam-10.1177_17588359251317842 – Supplemental material for Radiation-associated angiosarcoma of the breast: radical resection technique of the entire radiation field Supplemental material, sj-docx-1-tam-10.1177_17588359251317842 for Radiation-associated angiosarcoma of the breast: radical resection technique of the entire radiation field by Johannes Tobias Thiel, Michael Bauer, Adrien Daigeler, Vladyslav Kavaka, Jonas Kolbenschlag, Dominik Steiner, Anna Storz and Sebastian Hoffmann in Therapeutic Advances in Medical Oncology

## Declarations

**Ethics approval and consent to participate:** The retrospective study was conducted in accordance with the Declaration of Helsinki and was approved by the institutional Ethics Committee of the University of Tübingen (440/2024BO2; July 17, 2024). A written declaration of consent was obtained from all subjects participating in the study and the published patient images.

**Consent for publication:** Not applicable.

**Availability of data and materials:** The datasets used and analyzed in the current study are available from the corresponding author upon request.
