# Supplementary material for: Radiation-associated angiosarcoma of the breast: radical resection technique of the entire radiation field
Source: Ther Adv Med Oncol. 2025 Feb 18;17:17588359251317842. doi: 10.1177/17588359251317842 (PMC11837069; doi:10.1177/17588359251317842)
Supplement: sj-docx-1-tam-10.1177_17588359251317842 – Supplemental material for Radiation-associated angiosarcoma of the breast: radical resection technique of the entire radiation field [file sj-docx-1-tam-10.1177_17588359251317842.docx]

**Table 1:** Surgical technique reporting checklist and standards taken from: The SUPER reporting guideline suggested for reporting of surgical technique; Hepatobiliary Surg Nutr. 2023 Aug 1;12(4):534-544. doi: 10.21037/hbsn-22-509. Epub 2023 Jan 10. PMID: 37601001; PMCID: PMC10432285.

| **Section/Item** | **Item** | **Recommendation** | **Page** |
| --- | --- | --- | --- |
| Background | 1 | Describe the background of the disease or condition (e.g., its definition, classification, clinical manifestations,  epidemiological characteristics, and natural history) | 2-3 |
| Rationale | 2 | (I) Describe the pros and cons of existing treatments for the disease or condition, including currently used single or  combined surgical techniques | 2-3, 18-20 |
|  |  | (II) Explain whether the proposed surgical technique is a novel or modified procedure, including whether any modifications  have been made to key devices or materials. If only a conventional surgical technique is used, a brief description should be  accompanied by a citation of a source which describes the surgical technique in detail |  |
| Objectives | 3 | State what objectives and challenges the proposed surgical technique will address. Introduce what the surgical technique  figure and video will cover | 3-4 |
| Classification | 4 | Classify the surgical technique, either by: (I) surgical approach: open, minimally invasive (e.g., thoracoscopic, robotic), or  hybrid; or (II) treatment goal: curative or palliative | 4 and 10 |
| Name | 5 | Report the names of all involved surgical techniques in the title or abstract. If the surgical technique is the focus of the paper,  also include “surgical technique” in the title | 1-2 |
| Setting | 6 | I) Report information or requirements of the surgical environment (e.g., the name of the hospital, the hospital grade such as  tertiary hospital, the degree of cleanliness, and whether the procedure must be performed in an operating theatre) | 3 |
|  |  | (II) List and provide details of any special surgical equipment, supplies, drugs, or software used (e.g., the manufacturer,  product model, quantity, dosage, route, duration, and parameters) |  |
| Operators | 7 | Provide information about the surgical team personnel, including their role (e.g., surgeon, anesthetist, nurse), learning curve  (e.g., the number of cases), and training needed if applicable | Not applicable |
| Recipients | 8 | Report detailed indications and contraindications  (I) Disease or condition: type, etiology, the location, shape and size of the lesion, etc. | 3-10, 16 and 17 |
|  |  | (II) Recipients: age, sex, clinical manifestations, disease stage and severity, comorbidities and related complications, surgical  history and relevant family history, preoperative tests, pre-intervention, and other factors pertinent to successful practice |  |
|  | 9 | Provide detailed generic information and preparations  (I) Generic information: de-identified demographic information, symptoms and signs, imaging findings, staging,  comorbidities, and relevant therapy history, etc. | 16 and 17 |
|  |  | (II) Preparations: cardiovascular, gastrointestinal and respiratory tract preparation, urinary catheterization, skin preparation,  blood product preparation, anesthetic procedure and management, and patient positioning, etc. |  |
| Surgical approach,  key anatomic  landmarks, and  adjacent structures | 10 | (I) Describe in detail how to establish the surgical approach (e.g., devices and equipment used, the position of the surgeons,  anatomic localization, and the incision type, length, size, depth, angle, and number) | 4-14 |
|  |  | (II) Describe the essential anatomic landmarks and adjacent structures, including areas, structures, blood vessels, and  nerves, etc. (e.g., “use the Louis angle between the sternal manubrium and the sternal body to find the second costal  notch”) |  |
| Intraoperative  monitoring | 11 | Describe intraoperative monitoring specifically related to the surgical technique (e.g., near-infrared spectroscopy in aortic  arch surgery) | 4-14 |
| Step-by-step  description | 12 | Include all relevant details of each operative step in a step-by-step manner along with both quantitative and qualitative  description  (I) Details may include the intraoperative findings, timeline, histomorphology, exposure of vital structures, extent of lymph  node dissection, determination of surgical margins, suture pattern (running suture or single stitches; spacing of stitches),  anastomosis, knot-tying, specimen handling, and devices/supplies/drugs/blood products used, etc. | 4-14 |
|  |  | (II) Note the operative time |  |
|  |  | (III) If a non-conventional maneuver was applied, specify the reason |  |
| Quality and  consistency | 13 | Describe tips and skills for ensuring surgical quality and consistency, especially for the key steps and any conditions or  variations that require uniform management (if applicable). For example, using standardized training, establishing quality  control teams, and organizing multidisciplinary consultations | 4-14, 18-20 |
| Safety | 14 | Describe tips and skills for ensuring safety. For example, how to prevent or deal with possible intraoperative complications  and emergencies, or when and how to undertake a surgical conversion | 4-14, 18-20 |
| Visualization | 15 | (I) Visualize the key steps in a step-by-step and self-explanatory manner. Consider using narrated video(s) and anatomic  illustration(s) with designated symbols and illustrated text  (II) The key information in item 12 should be visualized; it can either be presented as a stand-alone figure or embedded in  the video(s)  (III) Visualization of the key information in items 10, 13, and 14 is encouraged as appropriate  (IV) After peer review, add clips into the video(s) to present the video title, operator name, and operation date at the  beginning, and the informed consent and the ethical approval statements at the end | 14-18 |
| Evaluation | 16 | I) Define the criteria for success and failure, and evaluate the efficacy or effectiveness of the surgical technique from both  the technical aspect and the clinical outcome perspective (e.g., length of stay, improvements in short- and long-term  mortality, recurrence, survival time, and patient impairment)  (II) When possible, include the perspective of the patient (e.g., symptoms and signs, postoperative pain, and aesthetic  results) | 16-20 |
| Postoperative  monitoring | 17 | Describe in detail postoperative monitoring specifically related to the surgical technique (e.g., monitoring indicators, devices,  frequency or duration, examination, and nursing required) | 14-18 |
| Complication  prevention and  management | 18 | Report the possible or observed postoperative complications and their prevention and management, especially  complications that differ from those related to conventional techniques | 16-20 |
| Follow-up | 19 | (I) Report the details of follow-up visits, including pathway, frequency, duration, and indicators (e.g., pathway-“telephone  follow-up”; frequency-“radiological examinations every 3 months”; duration-“up to 3 years”; indicators-poor outcomes,  complications, quality of life, and unexpected events)  (II) If applicable, compare the information in item 19a with those of conventional techniques | 16-20 |
| Strengths, limitations,  and outlook | 20 | Discuss the main strengths and limitations of the surgical technique, and provide detailed suggestions for improvement and  future outlooks | 18-20 |
| Impact and cost | 21 | I) Summarize the key points and take-away lessons of the surgical technique and its impact in the clinical setting and on  society (e.g., the economic cost)  (II) Consider in context the predominant cost and its potential impact on the implementation and adoption of the surgical  technique | 18-20 |
| Conflicts of interest,  ethical approval, and  informed consent | 22 | (I) Specify any potential conflicts of interest; (II) include the ethics committee or institutional review board approval (and the  number when applicable); and (III) provide the informed consent for publication | 21-22 |
